# Recent Advances in Salivary Scintigraphic Evaluation of Salivary Gland Function

**DOI:** 10.3390/diagnostics11071173

**Published:** 2021-06-28

**Authors:** Yen-Chun Chen, Hsin-Yung Chen, Chung-Huei Hsu

**Affiliations:** 1 Department of Otolaryngology, Taipei Medical University Hospital, Taipei 110, Taiwan; cd30617@gmail.com; 2 Department of Nuclear Medicine, Taipei Medical University Hospital, Taipei 110, Taiwan; 134004@h.tmu.edu.tw; 3 Division of Endocrinology and Metabolism, Department of Internal Medicine, Taipei Medical University Hospital, Taipei 110, Taiwan

**Keywords:** salivary scintigraphy, functional evaluation, visual analysis, quantitative, Sjögren’s syndrome

## Abstract

Saliva plays an important role in supporting upper gastrointestinal tract function and oral well-being. Salivary dysfunction mainly manifests with a decrease in salivary flow. Among varieties of quantitative methods, salivary scintigraphy is a relatively noninvasive, well-tolerated, reproducible, and objective approach for functional evaluation of salivary disorders, yet the lack of precise quantitative reference values and no standardized protocol limit its generalized utilization. In this article, we review the scintigraphic performance between the visual analysis and quantitative methods in predicting Sjögren’s syndrome and verify the potential aspects of the application in interpreting different disease entities and phases of functional salivary disorders.

## 1. Background

Salivary glands are responsible for the well-being of the oral cavity because saliva helps lubrication, enhances the taste and digestion of food, and maintains the integrity of teeth. Salivary gland dysfunction may manifest as painful swelling, thick or purulent discharge, or dry mouth (“xerostomia”). The prevalence of xerostomia, according to two longitudinal epidemiologic studies, is between 15.5% and 29.5%, and both studies supported the relationship between aging and the increased incidence of dry mouth [1,2]. Clinically, the degree of dry mouth may vary from transitory inconvenience to severe oral dysfunction with resultant psychosocial morbidities [3]. Although weak to no correlation between subjective oral dryness and salivary flow rates was observed [4], symptoms of dry mouth often appeared when unstimulated salivary flow decreased by 50% [5].

In typical clinical practice, in addition to the distinct signs of dry mouth (which Jager et al. simplified into a clinical oral dryness score [6] for rapid screening), various quantitative tests of salivary excretion have been proposed to evaluate dry mouth and can be divided into the following five categories, as reported by Löfgren et al. [7]:

(1) Secretion tests, including sialometry, the oral Schirmer’s test [8], the Saxon test [9], and methods that explores changes in salivary composition [10];
(2) Mucosal/surface tests such as measurements of mucosal saliva thickness [11] or salivary smears [12], or measurements of the impedance of oral mucosa on a moisture-checking device [13];
(3) “Functional” tests performed by using the dissolution of candy [14] or wafers [15];
(4) Glandular morphology, including sonography [16], magnetic resonance imaging (MRI) [17], sialography, and salivary gland scintigraphy (sialoscintigraphy);
(5) Questionnaires, interviews, or a combination of both [18].

Recently, Goto et al. compared the oral moisture level by using an electronic checking device, along with unstimulated and stimulated whole saliva volume in both young (mean age, 29.0 ± 5.4 years) and elder (mean age, 74.7 ± 5.9 years) volunteer groups by calculating the intraclass correlation coefficients for test–retest reliability, and they concluded that no consistent and reliable screening test for assessing salivary flow rate and oral dryness exists [19]. Although the method of spitting saliva for a period of time to measure the whole salivary flow rate was found to be more reliable and reproducible [19], patients who receive nursing care and those with cognitive impairment may be unable to repeatedly spit. Consequently, a basic question arises of whether any objective, reliable, and easy-to-perform method of measuring salivary flow exists. Among the current quantitative methods, sialoscintigraphy is a relatively noninvasive, well-tolerated, and objective approach to functionally and morphologically evaluate salivary disorders. The exam is cost effective for most hospitals that have a nuclear medicine department. However, the lack of precise quantitative reference values and standardized protocol, and the test’s limited ability to provide precise anatomic evaluations have resulted in the underutilization of sialoscintigraphy.

## 2. Mechanism and Procedure of Sialoscintigraphy

Sialoscintigraphy is a nuclear diagnostic imaging technique that assesses the major salivary gland function by using the radioactive tracer Technetium-99m pertechnetate (^99m^TcO_4_^−^) to measure glandular uptake and excretion. The sodium/iodide symporter (NIS), which is expressed by salivary gland epithelial cells and thyroid follicular cells [20], concentrates univalent anions such as Cl^−^ and I^−^. After it is administered, ^99m^TcO_4_^−^ can be actively concentrated by the major salivary glands and thyroid gland through the NIS in a manner similar to that of concentrating Cl^−^. This phenomenon reflects intact salivary gland parenchyma. The gathered anions in the salivary gland are then secreted into saliva, which indicates the glandular excretory function [21].

**Figure 1:**
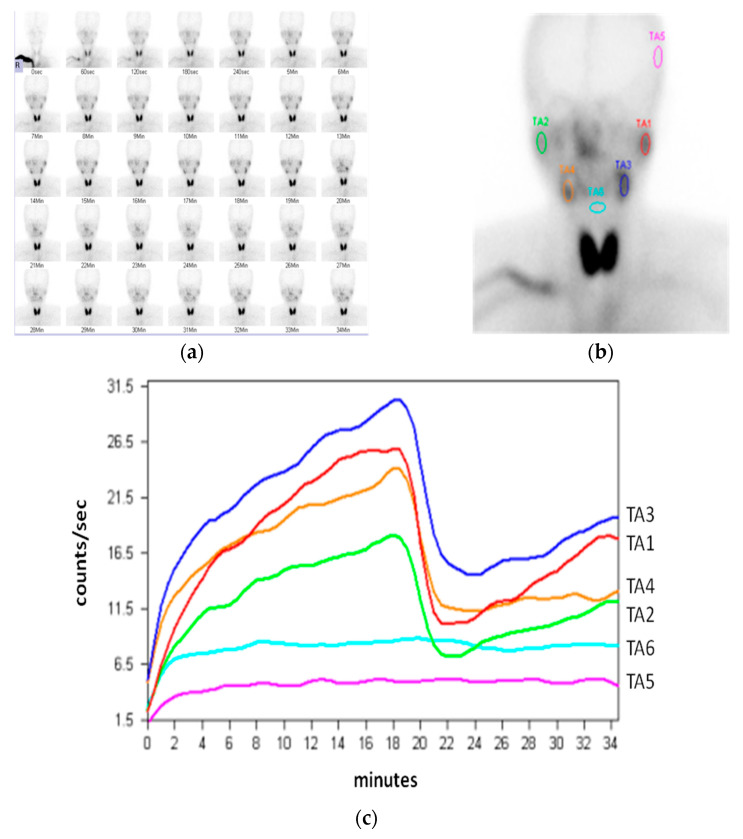
(**a**) Sequential imaging in sialoscintigraphy; (**b**) region of interest (ROI) positioned at the salivary glands, left temporal region of skull, and hypopharynx, respectively; TA1: left parotid; TA2: right parotid; TA3: left submandibular; TA4: right submandibular; TA5: temporal region as the background of the parotid gland; TA6: hypopharynx as the background of the submandibular gland; (**c**) time–activity curves (TACs) generated from six ROIs.

In our facility, individuals scheduled to undergo sialoscintigraphy were instructed to fast for more than two hours before the examination [22]. After 259 MBq (7 mCi) of ^99m^TcO_4_^−^ injected intravenously, immediate sequential images were acquired at the rate of one frame per 30 s for up to 70 frames, using a large field-of-view gamma camera equipped with a low-energy, high-resolution, parallel-hole collimator. At the 20th minute after injection, subjects were instructed to swallow 20 mL of diluted lemon juice, and total image recording was completed at the 35th minute (Figure 1a). On the summation image, regions of interest (ROI) with equal dimensions were set over the bilateral parotid and submandibular glands, and background regions (Figure 1b) to generate time–activity curves (TACs) (Figure 1c).

According to the thesis by ven der Akker in 1988 [23], a series of 17 normal subjects receiving sialoscintigraphy revealed the mean time of maximal ^99m^TcO_4_^−^ uptake for the submandibular glands and the parotid glands, which were 9 min (range 4–24 min) and 28 min (range 13–48 min), respectively. The thesis further denoted that several articles observed the mean time of maximal ^99m^TcO_4_^−^ accumulation for submandibular glands laid within the range of 9–14 min. Furthermore, in 2001, Aung et al. [24] demonstrated the time–activity curves of a 60-year-old healthy man and revealed that the bilateral submandibular curves showed spontaneous excretion at around 20 min. As a result, the period of 20 min was chosen as a prestimulatory observation period for the protocol in our facility.

The categorical classification pattern proposed by Schall et al. [25] in 1971, which is based on the degree of glandular uptake and isotope excretion into the oral cavity, is likely the most widely used method for interpreting sialoscintigraphic images [26], and abnormal results were adopted in the American–European Consensus Group (AECG) criteria for diagnosing Sjögren’s syndrome (SS) [27]. However, the visual assessment tends to be observer dependent [28] and limited in detecting borderline glandular dysfunction in early SS [29], and the activity in the oral cavity may be interfered with glandular spontaneous excretion and saliva swallowing during the acquisition period [30].

In the past, the value of salivary gland uptake divided by thyroid gland uptake, in addition to qualitative visual analysis, was usually used in the functional evaluation of salivary glands by using scintigraphy [31,32,33]. However, thyroid uptake may be influenced by several extrathyroidal conditions, as well as interference, environment, and subclinical thyroid diseases, or underlying autoimmune disorders [34]. The development of digital computing has enabled more unbiased assessments to be conducted using multiple quantitative methods [24,35,36,37] based on the time–activity curve. Various sets of quantitative indices have been devised to diagnose SS and classify its severity. However, wide dispersion in the normal values of most quantitative indices was found; this phenomenon possibly resulted from the heterogeneity of study populations and a lack of standardized algorithms for quantitative sialoscintigraphy, making the generalized utilization of quantitative assessment controversial [29].

## 3. Comparing Quantitative with Visual Evaluations

In past decades, although a quite variety of studies verifying the utility of quantitative methods in interpreting salivary scintigraphy in patients with sicca have been published and been concisely listed [30,38], the use of quantitative indices remains insufficiently supported. Two studies have directly compared the diagnostic performance of visual and semiquantitative sialoscintigraphic analyses when doctors had a clinical suspicion that a patient had SS [39,40]. Kim et al. assessed the sialoscintigraphic images of a total of 145 patients through a three-scale visual analysis and the quantitative indices as uptake ratio and percentage excretion. Of the 145 patients, 76 (52.4%) were diagnosed as having SS according to AECG criteria, and the remaining 69 (38 with fibromyalgia and 31 with isolated sicca) were assigned to the non-SS group. A relatively high sensitivity of 88.2% and a low specificity of 48.6% were found for the visual assessment method used to diagnose SS; these findings were consistent with those of previous studies [41] and were probably due to various etiologies of salivary disease having similar presentations. When the area under the ROC curve (AUC) of semiquantitative and visual analyses were compared, comparable results were found in both parotid uptake and excretion and submandibular uptake. However, the AUC for bilateral submandibular excretion was significantly lower than that of visual analysis. These findings led to the conclusion that the diagnostic ability of visual assessment was greater than that of semiquantitative assessment in diagnosing SS, especially for the submandibular glands. Moreover, the authors also found significant disagreement between visual and semiquantitative analysis that uses cutoff values for the presence of abnormalities in salivary glands.

In another study, recently Garcia-Gonzalez et al. [40] reviewed 137 patients with suspected SS and compared their salivary scintigraphic findings, which were obtained by using Schall’s classification grades and calculating the excretion fraction (EF%) for each gland. Based on a rheumatologist’s judgment, 54 patients (39.4%) were diagnosed as having SS, whereas the remaining 83 patients (44 with other autoimmune disorders and 39 with nonautoimmune sicca) were assigned to the non-SS group. Visual analysis revealed results similar to those of the study conducted by Kim et al. [39]. Both studies revealed that uptake dysfunction was more frequent than excretory dysfunction in the SS group; these findings implicated that more profound disease severity of SS might exist in both studies while taking the phenomenon into consideration that the earliest and most common scintigraphic abnormality observed in SS is impairment of excretion, followed by a decrease in tracer accumulation [42]. The AUCs of visual or submandibular EF%-derived parameters for utilizing AECG criteria in diagnosing SS were all significant. However, when Schall’s classification was used as a reference category for comparing SS diagnostic capability, no statistical differences were found between qualitative and quantitative scintigraphic analysis methods. When cutoff points from the ROC curves of the AECG criteria diagnostic modality were considered, the sensitivity and specificity for a cutoff of Schall’s grade ≥III were 68% and 84%, respectively, and for a submandibular EF% mean of <38, the sensitivity and specificity for the same cutoff were 73% and 59%, respectively. The authors concluded that although both the visual and EF%-derived index interpretation modalities were highly correlated with laboratory and pathophysiological features of SS, the highest performances of EF%-derived parameters in this study were only moderate and not superior to those of a Schall’s grade ≥III.

The results of the aforementioned studies justify the quantitative salivary scintigraphic assessments’ ability to discriminate SS, compared with that of visual methods, which might frustrate some researchers. Some authors have identified a wide dispersion for most quantitative indices [36,38,43], resulting in a large overlap in these parameters between the SS-positive and non-SS control groups. In our previous report [22], several parameters of the SS group and the reference values obtained from the asymptomatic glands of patients with obstructive sialadenitis also overlapped. The wide range of these parameters may be derived from widespread normal values, which was indicated by Ericsson and Hardwick [44] that resting and stimulated salivary secretion in healthy people could be categorized into the following three groups: normal secretors, low secretors, and very low secretors. Sreebny [45] further confirmed that although salivary flow rates varied widely between individuals, each individual’s salivary flow tended to remain reasonably consistent, and the mean flow rate for each person, rather than the mean flow rate for the population, was key. Furthermore, although semiquantitative indices, such as maximal accumulation or maximal excretion, refers to the percentage of tracer absorbed or excreted instead of the absolute count such as salivary flow rate, whether the proportion of isotope concentration and excretion varies widely between individuals or not remains largely unknown.

Currently, no consensus regarding which quantitative parameters are more trustworthy for diagnosing SS has been reached. Although some studies have demonstrated a preference for indicators based on excretion, especially the percentage of stimulated excretion fraction (EF%) [38,46], other studies [26,37] have found EF% to be less useful and have claimed that parameters associated with tracer uptake were more appropriate for diagnosing SS. Moreover, the possibility of bilateral glands having asymmetric involvement in SS [39,40] may interfere with the interpretation of test results. In both aforementioned studies that compared visual and semiquantitative methods of diagnosing SS, uptake dysfunction was more prevalent than excretory dysfunction in the SS group; this finding may indicate a more severe or advanced disease stage. This can result in more distinct changes in sialoscintigraphic images, and such changes might be easier to visually interpret. However, as Ramos-Casals et al. [41] noted, decreased uptake and delayed excretion of ^99m^TcO_4_^−^ is a nonspecific phenomenon that occurs in a variety of salivary disorders. The purpose of salivary scintigraphy is to quantitatively reflect the current status of the exocrine glands through uptake and excretory function in patients with SS or non-SS sicca, rather than to differentiate between SS and other conditions.

In addition to comparing the diagnostic capabilities of visual and quantitative methods, sialoscintigraphy can provide functional information on the major salivary glands and, improve clinicians’ confidence regarding current glandular status, thereby supporting subsequent therapeutic decisions with patients. Furthermore, the straightforward quantitative parameters can be implemented to help clinicians discriminate borderline conditions and identify early SS, especially in patients who require scintigraphic results because other AECG criteria are inconclusive, as Kaldeway et al. [38] indicated, since the salivary scintigraphy is sensitive enough to detect dysfunction caused by merely 25% parenchymal damage [29]. Additionally, scintigraphic measurements can be used to assess the therapeutic effects of interventions, such as the effects of sialendoscopic lithotripsy on patients with sialolithiasis or the impact of salivary intraductal irrigation on patients with chronic sialadenitis. Moreover, Ramos-Casals et al. [41] found that in patients with SS, the severe scintigraphic result at diagnosis was correlated with a higher risk of developing systemic features and lymphoma, and a lower survival rate, prompting a suggestion to repeat sialoscintigraphic exams every 2–3 years to follow up with patients and determine the outcomes of primary SS.

## 4. Applications of Sialoscintigraphy to Different Salivary Functional Disorders

The clinical impact of salivary scintigraphy has been reported in multiple functional salivary disorders, such as Sjögren’s syndrome, obstructive sialadenitis with or without parenchymal destruction [47], and iatrogenic irradiation-related sialadenitis due to the radiotherapy for head and neck tumors [48] or radioiodine (or I-131) treatment for thyroid cancers [49,50]. Zhang et al. [47] demonstrated that for 12 patients with larger (>5mm) or multiple parotid sialolithiasis, who were believed to have had less glandular recovery after lithotripsy, postoperative scintigraphy revealed notable partial functional improvement, although the function of the affected glands was still considerably lower than that of the contralateral control side. As functional restoration may occur in patients who have more severe obstructive sialadenitis, the following measures were also recommended: long-term follow-up, self-massage, and periodic intraductal irrigation with saline or steroid.

Regarding radiation-induced salivary gland injuries in patients with head and neck cancer who receive radiotherapy, scintigraphy plays a role in monitoring the progression of the irradiation effect, predicting the dose and volume relationships [48], and measuring the therapeutic response of radioprotective agents [51]. In addition to glandular uptake and excretory function, the morphology of the salivary glands and biofactors have also received attention for their role in evaluations of functional recovery. For example, Murdoch-Kinch et al. [52] reported that the salivary epidermal growth factor and other proteins in saliva returned to approximate preradiotherapy levels 12 months after patients received parotid-sparing radiation therapy. Regarding radioiodine-induced sialadenitis, Wu et al. [49] pointed out that the salivary gland scintigraphy of recipients of I-131 revealed a cumulative dosage-dependent association between I-131 and salivary gland dysfunction that primarily affected the parotid glands. A dose of up to 150 mCi did not affect uptake or excretory function, whereas a cumulative dose greater than 600 mCi resulted in the complete loss of excretion in the parotid glands. The common side effects of I-131 on salivary glands appear to be mild and transient; however, in recalcitrant chronic radioiodine sialadenitis, it seems that saline lavage through sialendoscopy followed with steroid instillation has a limited ability to relieve xerostomia and glandular dysfunction [53].

**Figure 2:**
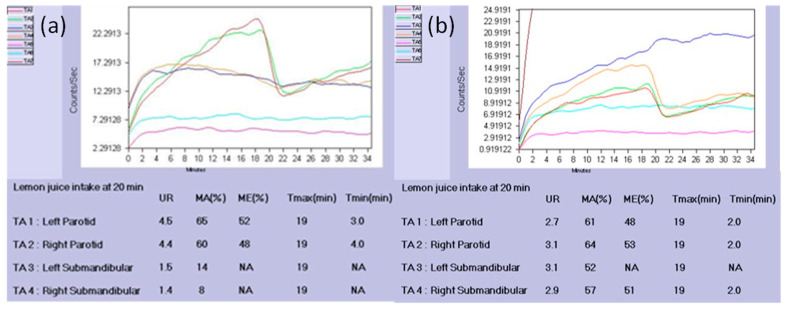
Time–activity curves (TACs) and semiquantitative parameters of (**a**) a patient diagnosed with SS, with totally diminished excretion (arrow); (**b**) a patient with sialolithiasis in left submandibular gland (arrow). UR: uptake ratio; MA: maximal accumulation; ME: maximal excretion; T_max_: time at the maximal count; T_min_: time interval from the peak activity point to the minimal count; NA: due to nearly no excretion detected from submandibular gland.

In the past decade, salivary gland ultrasonography has received more attention within the topic of SS diagnosis [17], and recently, a novel scoring system based on the percentage of anechoic/hypoechoic area ± the amount of normal surrounding tissue was released with good interobserver reliability and excellent intraobserver reliability [16]. Meanwhile, the use of salivary scintigraphy in the diagnosis of SS has continued to decline. Rather than evaluating glandular involvement with a morphological perspective, sialoscintigraphy provides functional assessment through numerical expression and requires a parenchymal dysfunction level of only 25% to identify SS. In 2019, our group found that scintigraphic data obtained from the unaffected glands of patients with single gland obstructive sialadenitis could be used as reference values for the evaluation of salivary disorders. The submandibular maximal excretion appeared to be the best indicator in distinguishing between the affected and unaffected glands in patients with obstructive sialadenitis, with an AUC of 0.82. Furthermore, when focusing on the submandibular glands, the maximal excretion in reference values revealed discriminating ability with the values in SS, with an AUC of 0.81 [22]. For example, in one patient who was diagnosed with SS, while the TAC (Figure 2a) of bilateral submandibular glands revealed a typical pattern of totally diminished excretion, the semiquantitative parameters further disclosed decreased tracer uptake, compared with the reference values. In another patient with sialolithiasis in the left submandibular gland, preoperative scintigraphic indices (Figure 2b) revealed comparable tracer accumulation with that of the contralateral unaffected gland, indicating a preserved parenchymal function in the obstructed side. These scintigraphic reference values were further used to predict the responsiveness of salivary intraductal irrigation with steroid in patients with chronic sialadenitis, although only the parameter as the total excretion time in the parotid gland was found to be positively related to the effect of irrigation in the SS group [54].

Despite the fact that salivary scintigraphy was withdrawn from the American College of Rheumatology–European League Against Rheumatism (ACR–EULAR) criteria for diagnosing SS [55], scintigraphic exams may be beneficial in distinguishing borderline conditions, following up with patients to determine disease progression, and aiding remedial decisions, especially when functioning glandular tissue demonstration supports the use of secretagogues as treatment. While the ability of quantitative analysis to diagnose SS was shown not superior to that of visual interpretation probably due to the paucity of consensus on which indices and a lack of standardized protocol, further multicenter studies are warranted to elucidate and repolish the role of salivary scintigraphy.

## Data Availability

No new data were created or analyzed in this study. Data sharing is not applicable to this article.
